# Dengue Virus Directly Stimulates Polyclonal B Cell Activation

**DOI:** 10.1371/journal.pone.0143391

**Published:** 2015-12-10

**Authors:** Arturo Ramon Vargas Correa, Ana Carolina Egypto Rosa Berbel, Michelle Premazzi Papa, Ana Theresa Silveira de Morais, Ligia Maria Torres Peçanha, Luciana Barros de Arruda

**Affiliations:** 1 Departamento de Virologia, Instituto de Microbiologia Paulo de Góes, Universidade Federal do Rio de Janeiro, Rio de Janeiro, RJ, Brasil; 2 Departamento de Imunologia, Instituto de Microbiologia Paulo de Góes, Universidade Federal do Rio de Janeiro, Rio de Janeiro, RJ, Brasil; University of Hong Kong, HONG KONG

## Abstract

Dengue infection is associated to vigorous inflammatory response, to a high frequency of activated B cells, and to increased levels of circulating cross-reactive antibodies. We investigated whether direct infection of B cells would promote activation by culturing primary human B lymphocytes from healthy donors with DENV *in vitro*. B cells were susceptible, but poorly permissive to infection. Even though, primary B cells cultured with DENV induced substantial IgM secretion, which is a hallmark of polyclonal B cell activation. Notably, DENV induced the activation of B cells obtained from either DENV immune or DENV naïve donors, suggesting that it was not dependent on DENV-specific secondary/memory response. B cell stimulation was dependent on activation of MAPK and CD81. B cells cultured with DENV also secreted IL-6 and presented increased expression of CD86 and HLA-DR, which might contribute to B lymphocyte co-stimulatory function. Indeed, PBMCs, but not isolated B cells, secreted high amounts of IgG upon DENV culture, suggesting that interaction with other cell types *in vivo* might promote Ig isotype switching and IgG secretion from different B cell clones. These findings suggest that activation signaling pathways triggered by DENV interaction with non-specific receptors on B cells might contribute to the exacerbated response observed in dengue patients.

## Introduction

Dengue viruses (DENV) belong to the *Flaviviridae* family and comprise four genetically distinct serotypes (DENV1-DENV4), responsible for millions of infections each year in tropical and subtropical areas of the world. According to the World Health Organization dengue incidence has highly increased over the past 50 years, turning this infection the most important arthropod-born disease in the world and a global health challenge [1, 2].

Dengue infection causes clinical manifestations ranging from mild to severe symptoms associated to fever, hemorrhagic manifestations, increased vascular permeability and plasma leakage, and may be a life threatening disease [3, 4]. Severe dengue is more common in secondary infections and it has been suggested that the activation of low-affinity cross-neutralizing T and/or B cells, and an exacerbated inflammatory response are correlated to disease severity [5, 6, 7, 8]. The most widely supported theory proposed to explain the increased risk of severe dengue is antibody dependent enhancement (ADE), which postulates that antibodies from previous heterologous infection are cross-reactive and poorly neutralize the circulating virus in a secondary episode [4, 9]. The immune complexes generated by these antibodies would then facilitate virus entry in FcR-bearing cells [10, 11]. In fact, a large fraction of antibodies generated during both primary and secondary infections are serotype cross-reactive and non-neutralizing, indicating that antibody response during dengue infection is very complex and may either benefit or harm the patient [12, 13, 14, 15, 16].

Activation of B lymphocytes may be triggered by antigen-specific BCR activation and/or by other polyclonally distributed receptors, including pathogen recognition receptors (PRRs), B cell coreceptor complex, and costimulatory receptors (e.g. CD40, BAFFR, among others). Effective antibody response depends on the integration of multiple signals that converge at the level of transcription factor activation, and induces B cell proliferation and differentiation into effector plasma cells or long lived memory B cells [17, 18, 19, 20, 21, 22]. Mitogen-activated protein kinases (MAPK), including extracellular signal-regulated kinase (ERK), c-Jun NH2-terminal kinase (JNK/SAPK) and p38 MAPK, are downstream mediators of signal transduction pathways targeted by some of the cited receptors, and their activation influence on nuclear translocation of transcription factors involved in B cell activation and survival [22, 23, 24, 25]. Intracellular signaling initiated by BCR can be potentiated by the activation of a co-receptor complex formed by CD19, CD21, CD81, and CD225, which decrease the threshold for BCR-dependent activation [26, 27, 28]. The signaling pathway stimulated by the activation of coreceptors is usually associated to phosphorylation of the cytoplasmic tail of CD19 [29, 30]; but previous studies have demonstrated that B cell proliferation and Ig somatic hypermutation can also be stimulated by CD81 engagement without the colligation of BCR or CD19 [31, 32].

Very recent studies start to unveil the events associated to the high incidence of cross-reactive antibodies during dengue infection. It was demonstrated that most of DENV-exposed persons present a high frequency of circulating plasma cells and plasmablasts, which is correlated with the appearance of cross-reactive IgG against other DENV serotypes and unrelated antigens [33, 34, 35]. These findings suggest that natural poly-reactive B cells may be directly stimulated by DENV infection. However, the effect of DENV interaction with B lymphocytes on B cell activation was not addressed yet. Here, we investigated whether DENV directly modulate human B cell activation and the signaling pathways associated to this stimulation.

## Methods

### Cells and virus

C6/36 *Aedes albopictus* mosquito cell line (kindly provided by Dr. Andrea T. Da Poian, IBqM, UFRJ) [36] were cultured at 28°C in Leibovitz (L-15) medium (Life Technologies, Grand Island, NY) supplemented with 10% of tryptose phosphate broth, 0.75% sodium bicarbonate, 0.2% of l-glutamine (Sigma-Aldrich, St Louis, MO), and 10% FBS (Life Technologies).

DENV serotype 2, Jamaica strain [37] or DENV1 (isolated from a patient; kindly provided by Dr. Maria Teresa V. Romanos, UFRJ) were propagated in the C6/36 cell line. After 7 days p.i., the supernatants were harvested, filtered and ultracentrifuged (110000g) to obtain a virus-enriched suspension. The stock virus titer was determined by observation of cytopathic effect and the TCID50 was calculated according to Reed & Muench [38]. Supernatants of noninfected C6/36 cells cultured in the same conditions were used as mock control. Inactivated virus (iDENV) was obtained after U.V. exposition for 2 hours and the inactivation was confirmed by RT-PCR in C6/36 cells.

### Ethics Statement

Blood samples (buffy coats) from healthy donors were obtained anonymously from the Hemotherapy Service from the Hospital Universitário Clementino Fraga Filho (HUCFF) of Universidade Federal do Rio de Janeiro (UFRJ). The study protocol was approved by the Experimental Ethics Committee of UFRJ (Permit Number: IMPPG 025) and the review board waived the need for informed patient consent.

### PBMC and B cell isolation

Peripheral blood mononuclear cells were obtained after centrifugation of buffy coats samples over ficoll-hypaque gradients. Purified B cells were isolated from the PBMCs by negative selection using a B-cell isolation kit II (Miltenyi Biotec, Bergisch-Gladbach, Germany), according to manufacturer's protocol. The purity of isolated B cells (CD19^+^) was higher than 93% as assessed by flow cytometry analysis. In some experiments, PBMCs were cultured for 2h for cell adhesion and nonadherent cells were stained with anti-CD19 or anti-CD3 and anti-CD27 antibodies (eBiosciences, San Diego, CA). CD19^-^ cells were sorted as non B cells; CD3^-^CD27^-^ cells were sorted as naïve B cells. Cell sorting was performed in a MoFlo equipment (Dako Colorado, Inc.) and sorting strategy is shown in S1 Fig. Naïve B cells were cultured either separately or with nonB cells, at the proportion 1:9. PBMCs or B cells were cultured with RPMI, supplemented with 10% FCS (Life Technologies) (complete medium) at 37°C in 5%CO_2_ atmosphere. Serum samples of each individual donor were screened for IgG antibodies against dengue virus by using an enzyme-linked immunoassay commercial kit (Dengue IgG-ELISA, PanBio, Ltd., Brisbane, Australia).

### Analysis of cellular infection by qRT-PCR

Purified B cells or C6/36 cell line were cultured at 24 well plates at 5 x 10^5^ cells/well and incubated with mock, DENV2 (MOI of 1) or iDENV2 (MOI of 1) for 2h at 37°C for virus adsorption. The cells were extensively washed with PBS and then cultured with the appropriate culture medium. Cells and supernatant were then harvested soon after virus adsorption (0h; input control) and after different time points from 24 to 72 hours. RNA Samples were extracted using TRIzol Reagent, according to manufacturer’s prococol (Life Technologies). In some experiments, C6/36 cell line were cultured with 200 μl of supernatants obtained from infected B cells and RNA from cells and supernatants were also extracted at the same time points.

The samples were treated with DNAse I (Fermentas) to prevent genomic DNA contamination and first strand cDNA was synthesized using 2 μg of the RNA extracted from mock-treated or DENV-infected cells using High-Capacity cDNA Archive Kit (Life Technologies, Applied Biosystems, Foster City, CA, U.S) For each sample, reactions were prepared with a final volume of 10 μL, containing RT buffer 1x, dNTP mixture 1x, 2,5U/mL of reverse transcriptase Multiscribe and Random primers 1x and RNA. The thermal cycling was performed on MicroAmp 9600 thermocycler (Applied Biosystems) programmed for 25°C for 10 minutes and 37°C for 120 minutes. cDNAs (5μl) obtained as described were subjected to quantitative real-time PCR for detection of viral RNA using a StepOnePlus Real-time PCR system (Life Technologies) and Taqman Master Mix Reagents (Life Technologies), as described elsewhere [38] Primers used were DEN-2 C (CCATCTGCAGCAACACCATCTT) and DEN-2 F (CAGGTTATGGCACTGTCACGAT) and the probe was 5’ /56FAM CTCTCCGAGAACAGGCCTCG. Reaction was held in the following conditions: 95°C for 10 minutes and 45 cycles of 95°C for 15 seconds, 45°C for 30 seconds and 72°C for 1 minute. The fluorescence readings were performed by 7500 Real-Time PCR equipment (Applied Biosystems) for each cycle of amplifications and then, analyzed by Sequence Detection Software (SDS) v1.3 (Applied Biosystems). Virus RNA concentration was determined, by using a standard curve, as previsouly described [36]

### Immunofluorescence and flow cytometry analysis

Purified B cells were infected as described and, after 48h, the cells were harvested, fixed with 2% paraformaldehyde, and permeabilized/blocked with 0.1% saponin and 2.5 μg of human Fc Block (BD Biosciences). The cells were then incubated with anti-DENV NS1 antibody (1:50; Abcam, Cambridge, MA) or with anti-DENV2 3H5 (ATCC hybridoma), followed by AlexaFluor conjugated anti-mouse IgG. For the IF assays, the cells were cultured onto coverslips, the fixation, permeabilization and stained were performed, and then the coverslips were stained with DAPI (Biolegend, San Diego, CA) and mounted onto glass slides using ProLong Antifade reagent (Molecular Probes, Eugene, OR). For the flow cytometry assays, the cells were acquired using FACSCalibur equipment and analyzed with FlowJo software (BD Biosciences).

### Analysis of Immunoglobulin secretion

PBMC or purified B cells (2 x 10^5^ cells/well) were cultured with DENV 2 or DENV 1, with a MOI of 1, for 2 h at 37°C in 5% CO_2_ atmosphere. Control cultures were performed using supernatant of noninfected C6/36 cells (mock-infected) or U.V.-inactivated DENV (iDENV). PWM (10μg/mL, Sigma) or Sac (1μg/mL, Sigma) were used as positive controls in the PBMC or purified B cells cultures, respectively. In some experiments, DENV-infected cells were also cultured in the presence or absence of MAPK inhibitors, including PD98059 (ERK inhibitor; 30μM), SB203580 (p38 inhibitor; 10μM) and SP600125 (JNK inhibitor; 10μM) (Cell Signaling Technology, Danvers, MA); or in the presence of anti-CD81 neutralizing antibody (1μg/mL; BD Biosciences), or anti-TLR4 neutralizing antibody (500ng/ml; Invivogen, San Diego, CA). After 12 days of culture, supernatants were harvested and IgM or IgG levels were analyzed by capture ELISA. Briefly, ELISA plates were incubated, overnight at 4°C, with anti-human IgM or anti-human IgG antibodies, at 3 μg/ml (Sigma Aldrich). The plates were blocked with PBS containing 10% FCS for 2 hours/37°C, washed with PBS-0.05%Tween 20 (PBS-T) and the supernatant samples were added, in serial dilutions, and incubated at 4°C/overnight. Purified human IgM or IgG were also added, at serial dilutions, in order to obtain a standard curve. The plates were incubated with alkaline phosphatase (AP)-conjugated anti-human IgM or IgG (1 μg/ml; Life Technologies) for 2 hours/37°C, washed and developed using pNPP substrate (Sigma Aldrich). The reaction was read at 405nm using an ELISA reader (BioRad Laboratories Inc., Hercules, CA).

### Cytotoxicity assays

To evaluate toxicity of the antibodies and pharmacological inhibitors, cells were cultured in the presence or absence of anti-CD81, PD98059 (30μM), SB203580 (10μM) or SP600125 (10μM) for 72h. Then, the cells were stained with propidium iodide (PI, 50μg/ml, BD Bioscience) and analyzed by flow cytometry, using FACSCalibur equipment and CellQuest software. To further confirm the data, the viability of B cells cultured with anti-CD81 were also assessed by XTT assay (Sigma Aldrich), according to the manufacturer’s protocol; and the concentration of lactate dehydrogenase (LDH) in culture supernatant were evaluated, using LDH assay Kit (Doles, Goias, Brasil).

### Western Blotting

B cells were mock-treated or cultured with native or inactivated DENV2, at a MOI of 1, in the presence or absence of anti-CD81 antibody. After different time points, from 2h to 48h, cells were lysed with low salinity buffer (10mM Tris-Cl pH 7,5; 5mM de EDTA pH 8,0; 150mM NaCl; 0,1% NP-40) for 30 minutes at 4°C. The homogenates were clarified by centrifugation at 13000 g at 4°C for 10 min, samples were resuspended in sample buffer (SDS 5x) being normalized according to the total protein concentration, using DC Protein Assay II kit (BioRad Laboratories Inc.). Samples and standard molecular weight were subjected to 12% SDS-PAGE and transferred to nitrocellulose membrane (Amersham Hybond—ECL, Ge Healthcare Life Sciences, Little Chalfont, U.K). The membranes were incubated with anti-phospho-p44/42 MAPK (Erk1/2) (1:2000), anti-phospho-p38 MAPK (1:1000), anti-phospho-SAPK/JNK (1:1000), anti-p44/42 MAPK (Erk1/2) (1:5000), anti-p38 MAPK (1:5000), anti-JNK (1:1000) (Cell Signaling Technology, Beverly, MA), anti-phosphotyrosine (4G10 clone; Merck Millipore, MA), anti-phospho AKT (1:200, Santa Cruz Biotechnology, Dallas, TX), anti-AKT (1:2000; Cell Signaling Technology), and anti-and β-actin (1:2000, Santa Cruz Biotechnology). After five washes with PBS-T, the membranes were incubated with HRP-conjugated anti-mouse or anti-rabbit IgG antibodies (1:5000). Super Signal^®^ West Pico Chemiluminescent Substrate (Thermo Scientific) was used for protein detection according to the manufacturer’s instructions. The ratio of phosphorylated/unphosphorylated protein was determined using ScionImage software.

### Analysis of IL-6 secretion

Purified B cells were cultured as described. After 48h of infection, supernatants were harvested and IL-6 was measured using Human IL-6 ELISA Ready-SET-Go! Kit (eBiosciences, San Diego, CA). Briefly, ELISA plates were incubated with anti-human IL-6 antibody (5 μg/ml), overnight at 4°C. The plates were blocked with 1x Assay Diluent for 1hour/RT, washed with PBS-T, the supernatant samples were added in serial dilutions, and incubated at 4°C/overnight. Recombinant human IL-6 was also added, at serial dilutions, in order to obtain a standard curve. The plates were, then, incubated with biotin-conjugated anti-human IL-6 antibody (5 μg/ml) for 1 hour/RT, washed and incubated with HRP-conjugated avidin for 30 minutes at RT. After several washes with PBS-T, the plates were developed using TMB substrate. After 15 minutes, the reaction was stopped with 1 M H_2_SO_4_ and read at 450nm using an ELISA reader (BioRad Laboratories Inc.).

### Analysis of B cell phenotype

Purified B cells were mock-treated or incubated with DENV2 (MOI = 1) for different time points p.i., from 3 to 9 days. After that, the cells were harvested, fixed with 4% paraformaldehyde, blocked with Fc-block, and incubated with anti-CD19-FITC and anti-CD86 or anti-HLA-DR conjugated to PE (1μg/mL) (eBioscience, Inc., San Diego, CA). The expression of CD86 or HLA-DR among CD19+ cells was evaluated by flow cytometry. 10000 events were acquired and the analyses were performed using cell quest software.

### Statistical analysis

The mean and SD were calculated for each experimental group. Differences between groups were analyzed by the *Student t* test for unpaired samples using the PRISM statistical analysis software (GraphPadSoftware, Inc., San Diego, CA). Results with p < 0.05 were considered significant.

## Results

### B cells are susceptible, but poorly permissive to DENV infection

**Fig 1:**
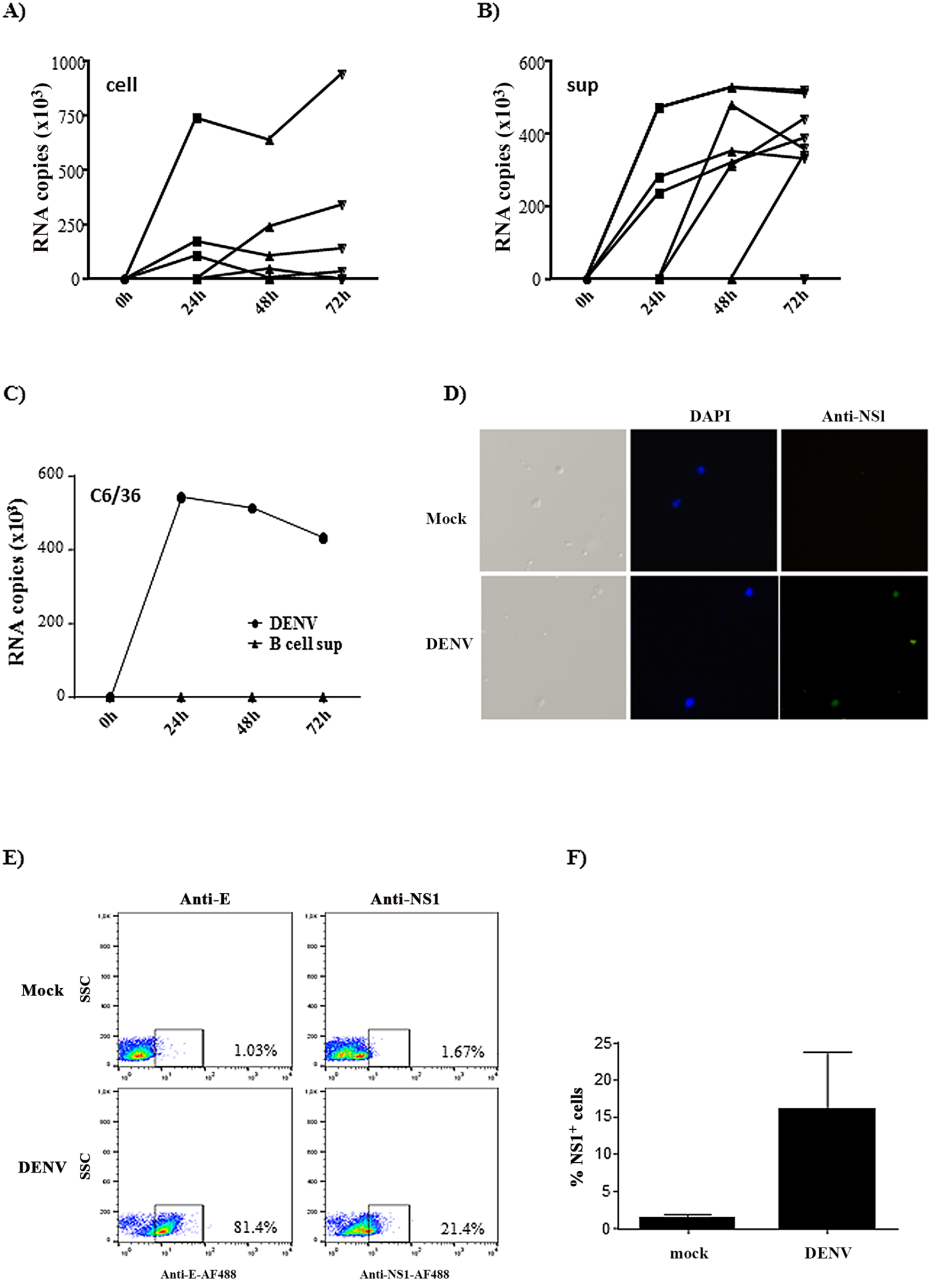
B lymphocytes are susceptible, but poorly permissive to DENV infection. A-B) Purified B lymphocytes were mock-treated or cultured with DENV2 (MOI = 1) for the indicated time points. Cell lysates (A) and supernatants (B) were harvested and virus RNA was measured by quantitative real time RT-PCR. Each line indicate an individual donor after normalization by subtracting the input values. C) C6/36 cell line was incubated with DENV2 (MOI = 1) or with supernatants obtained from B cells previously cultured with DENV. After the indicated time points the cells were harvested and virus RNA was measured by qRT-PCR. D) Purified B cells were mock-treated or incubated with DENV2. After 48h, the cells were incubated with anti-NS1 antibody, followed by anti-mouse IgG conjugated to AlexaFluor488, and DAPI. The expression of DENV NS1 was analyzed by fluorescence microscopy. E-F) Purified B cells were cultured as in *D* and stained with anti-3H5 or anti-NS1 antibodies. A representative dot blot is shown in E and the average of the percentage of NS1^+^ cells obtained from 3 individual donors is shown in F.

B cell infection by dengue virus was not completely established; therefore, we initially investigated whether human primary B lymphocytes would be productively infected by the virus in an *in vitro* system. Cells from 10 individuals were cultured with DENV2 and the amount of virus RNA in the cells and supernatants were analyzed at different time points post infection (p.i.) by real time quantitative PCR. Seven out of ten donors expressed DENV RNA. In most samples virus RNA was detected after 24 hours p.i. in the cell and supernatant fractions; however, cDNA levels showed a slight or no increase in the subsequent time points (Fig 1A and 1B). This data suggest that the virus was internalized and released by B cells, but the secreted viruses might be inefficient to further infect the cells. To confirm this hypothesis, we incubated C6/36 cells with the supernatants obtained from DENV-infected B cells and analyzed the infection by qRT-PCR. In fact, DENV RNA was not detected in C6/36 cells at any time point analyzed (Fig 1C). B cell infection was also evaluated by immunofluorescence and FACS, after staining with anti-E or anti-NS1 antibodies (Fig 1D–1F). We observed around 80% and 20% of B cells expressed E and NS1 protein, respectively (Fig 1E), suggesting that B cells were highly susceptible to DENV infection, although not all the particles completed the replication cycle. Taken together, these data suggest that B cells were actually infected by DENV, but the new virus particles produced might be mostly immature or defective.

### DENV induces immunoglobulin secretion by primary human B cells

**Fig 2:**
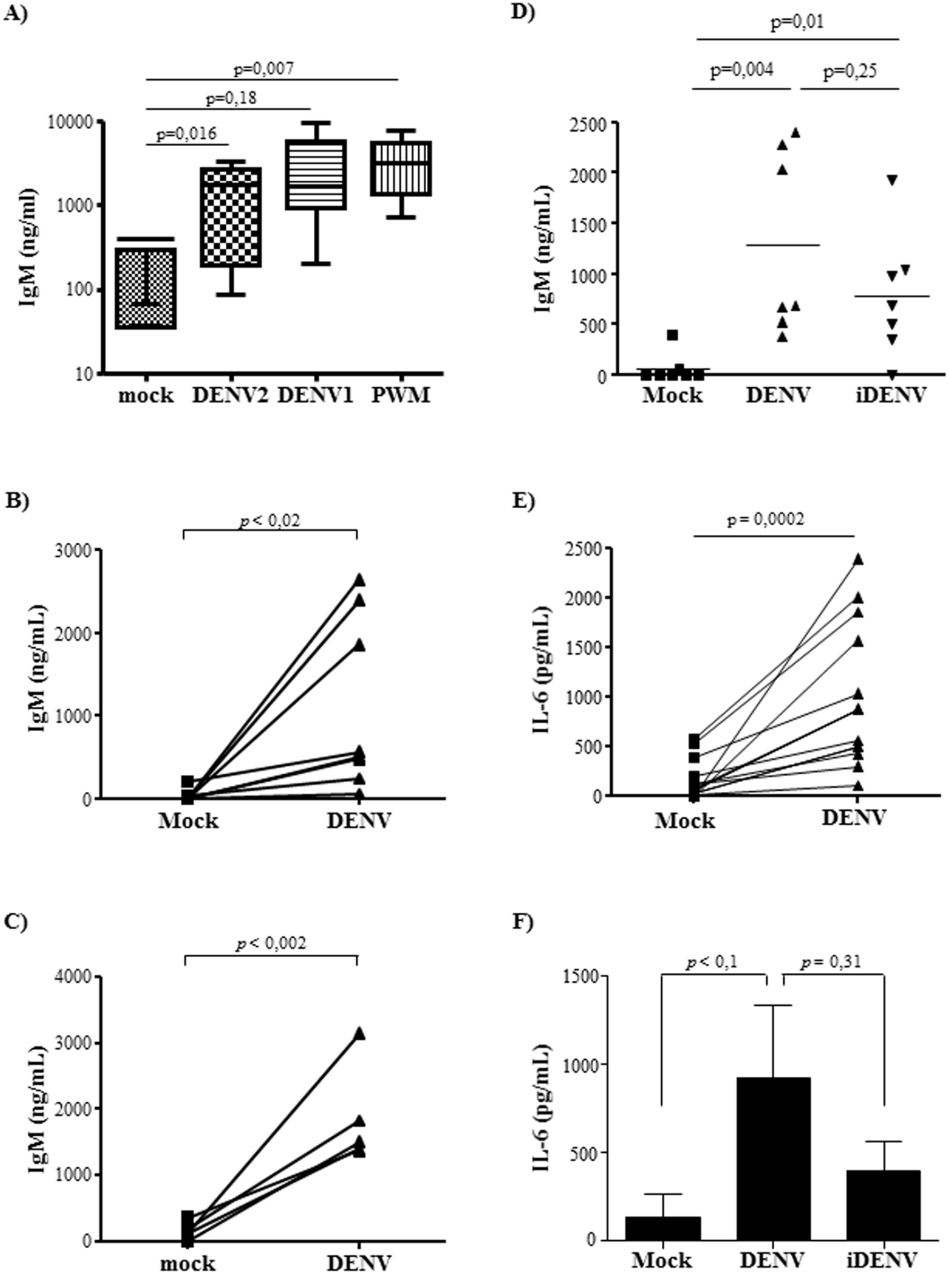
DENV induces polyclonal B cell activation. **A)** PBMCs isolated from healthy donors were mock-treated or cultured with DENV2 or DENV1, at an MOI of 1. PWM was added in the cultures as a positive control. After 12 days, the supernatants were harvested and total IgM levels were measured by ELISA. The graph indicates the median and standard deviation of 6 individual donors **B-C)** Purified B lymphocytes obtained from DENV seropositive **(B)** or seronegative **(C)** donors were mock-treated or cultured with DENV2 at a MOI of 1. After 12 days p.i., the supernatants were harvested and IgM levels were measured by ELISA. The lines indicate the IgM response to mock-treatment or DENV infection obtained from the same donor. **D)** The cells were mock-treated or cultured with DENV2, either native or inactivated by U.V. radiation (iDENV). After 12 days p.i., IgM supernatant levels were measured by ELISA. Individual donors are showed in the graph. **E)** B lymphocytes were mock-treated or cultured with DENV2 (MOI = 1), supernatants were harvested after 48h p.i. and IL-6 levels were measured by ELISA. The lines indicate the IgM response to mock-treatment or DENV infection obtained from the same donor. **F)** The cells were mock-treated or cultured with DENV2, either native or inactivated by U.V. radiation (iDENV). After 48h p.i., IL-6 supernatant levels were evaluated by ELISA. Bars indicate the average and standard deviation of five individual donors. Statistical analysis were performed and p values are indicated in the figures.

DENV-infected individuals present an increased frequency of plasmablasts correlated to high amounts of cross-reactive DENV-specific immunoglobulin in their sera. This suggests that virus infection results in B cell activation and stimulation of Ig secretion. To evaluate whether the virus itself could induce B lymphocyte activation, we isolated cells from healthy donors that have experienced or not DENV infection (based on DEV-specific IgG serum levels) and infected with DENV. PBMCs cultured with DENV2 or DENV1 showed a significant increase in IgM secretion, induced by either virus serotype (Fig 2A). We then investigated whether this activation could be mediated by a direct contact between the virus and B lymphocytes, and whether B cell activation was dependent on a productive infection. CD19+ B cells were purified from PBMCs and both DENV1 (S2 Fig) and DENV2 (Fig 2B–2D) induced great amount of IgM secretion. Importantly, significant increase in IgM secretion was observed in the cultures performed with B lymphocytes from either DENV immune (Fig 2B) or DENV naïve (Fig 2C) donors, indicating that activation of B cells was not only associated to a memory, recall response. B cells cultured with U.V-inactivated DENV (iDENV) showed slightly lower levels of IgM secretion, in comparison to the levels induced by native virus, but it was still significantly different from control cultures (Fig 2B). These findings suggests that interaction of DENV structural proteins with B cells receptors could trigger a signaling cascade that culminates with induction of immunoglobulin secretion.

Since earlier studies demonstrated that DENV infection induced IL-6 secretion by human B cells *in vitro* [39], we analyzed whether this cytokine was also being secreted in our system. IL-6 was detected in DENV-stimulated cultures (Fig 2E) and, similar to what was observed with IgM secretion, cytokine secretion induced by inactivated virus was lower, but not statistically different than the one induced by native DENV (Fig 2F).

### B cell activation by DENV depends on MAPK signaling phosphorylation

**Fig 3:**
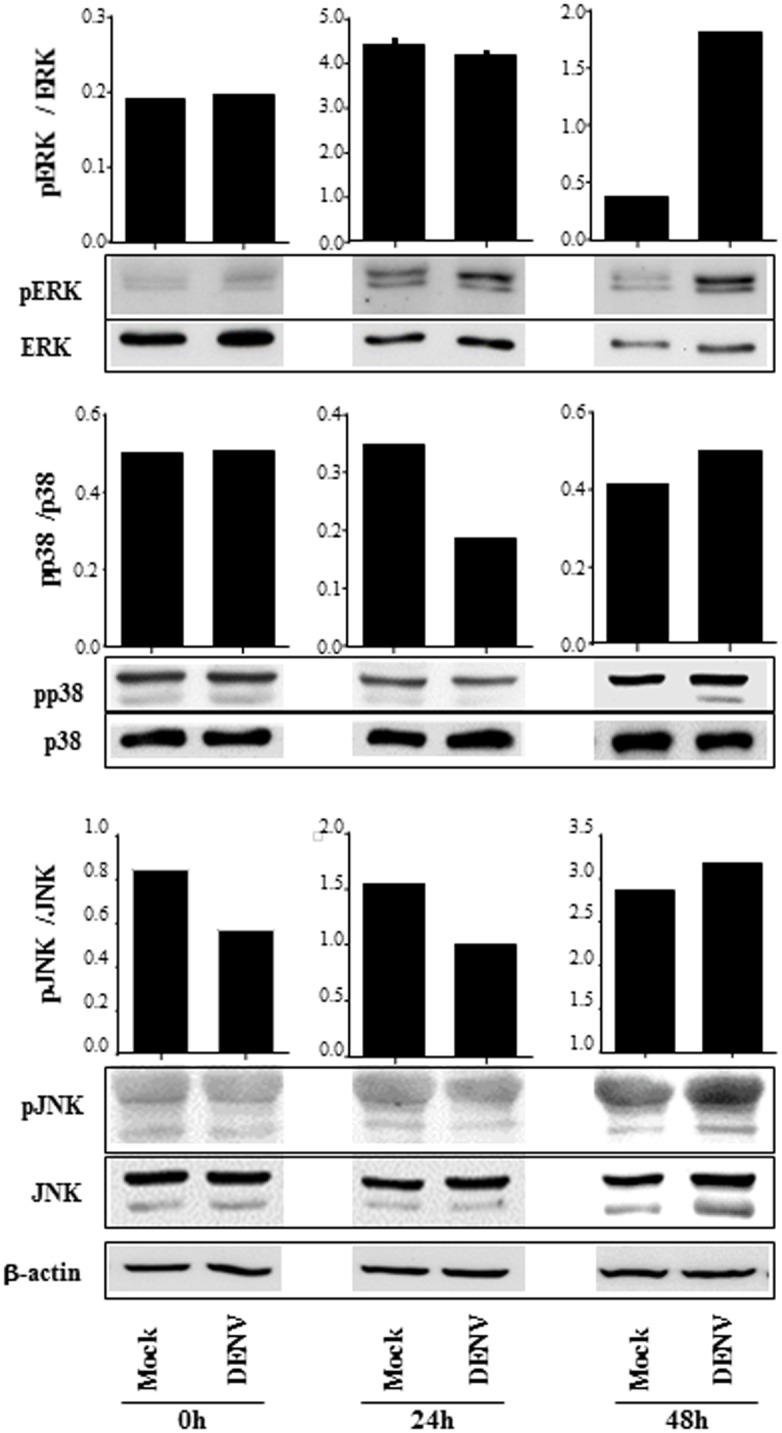
B cell infection by DENV promotes MAPK phosphorylation. B lymphocytes were mock-treated or cultured with DENV2 (MOI = 1). The cells were harvested after 2h, 24h or 48h p.i., and the expression of ERK, p38 and JNK MAPK were analyzed in the cell lysates by western blotting, using the indicated antibodies. The bars indicate the ratio between the analyzed phosphorylated protein and the corresponding non phosphorylated one; β actin staining were performed as a loading control and is shown in the bottom of the figure. Data are representative of three independent experiments.

**Fig 4:**
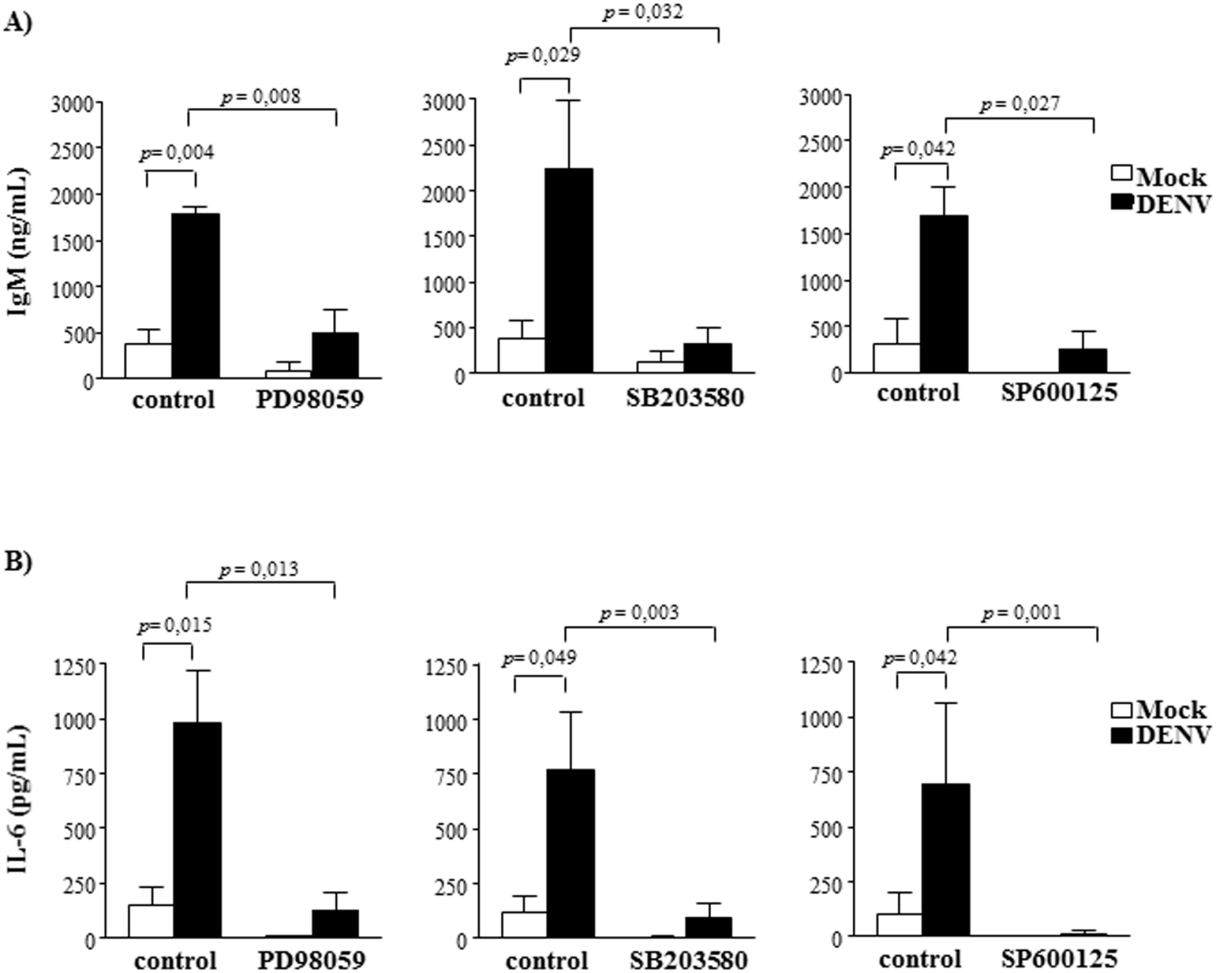
Activation of MAPK is essential for DENV-induced B cell activation. Mock or DENV-treated cells were cultured in the presence or absence of ERK (PD98059), p38 (SB203580) and JNK (SP600125) inhibitors. **A)** After 12 days culture, supernatants were harvested and IgM levels were measured by ELISA. **B)** After 48h, culture supernatants were harvested and IL-6 levels were measured by ELISA. Data are representative of at least four independent experiments. Statistical analysis were performed and p values are indicated in the figures.

B cell activation induces tyrosine or serine-threonine phosphorylation of adaptor molecules, leading to stimulation of different signaling cascades, including PI3K and MAP kinases (MAPK) signaling pathways. To analyze which pathways were involved in DENV-mediated B cell activation, we evaluated the phosphorylation pattern of distinct signaling molecules in DENV-infected cultures. Western blotting analysis of total tyrosine phosphorylation pattern showed no difference between mock-treated and DENV-infected B lymphocytes. Similarly, we did not detect increased AKT phosphorylation, which would be an indicative of PI3K activation (S3 Fig). In contrast, increased phosphorylation levels of ERK, p38 and JNK MAPK were observed when purified B cells were cultured with DENV, in contrast to mock-treated cultures (Fig 3). In order to confirm the importance of these signaling pathways in DENV-induced B cell activation, the cells were infected in the presence or absence of specific inhibitors of each MAPK, and IgM and IL-6 secretion were evaluated by ELISA. Supernatant IgM and IL-6 concentrations were drastically reduced when the cells were cultured with all the analyzed inhibitors (Fig 4A and 4B), indicating that activation of ERK, p38and JNK MAPK were essential for the activation of B cells by DENV.

### DENV-induced IgM secretion is independent on TLR4, but dependent on CD81

**Fig 5:**
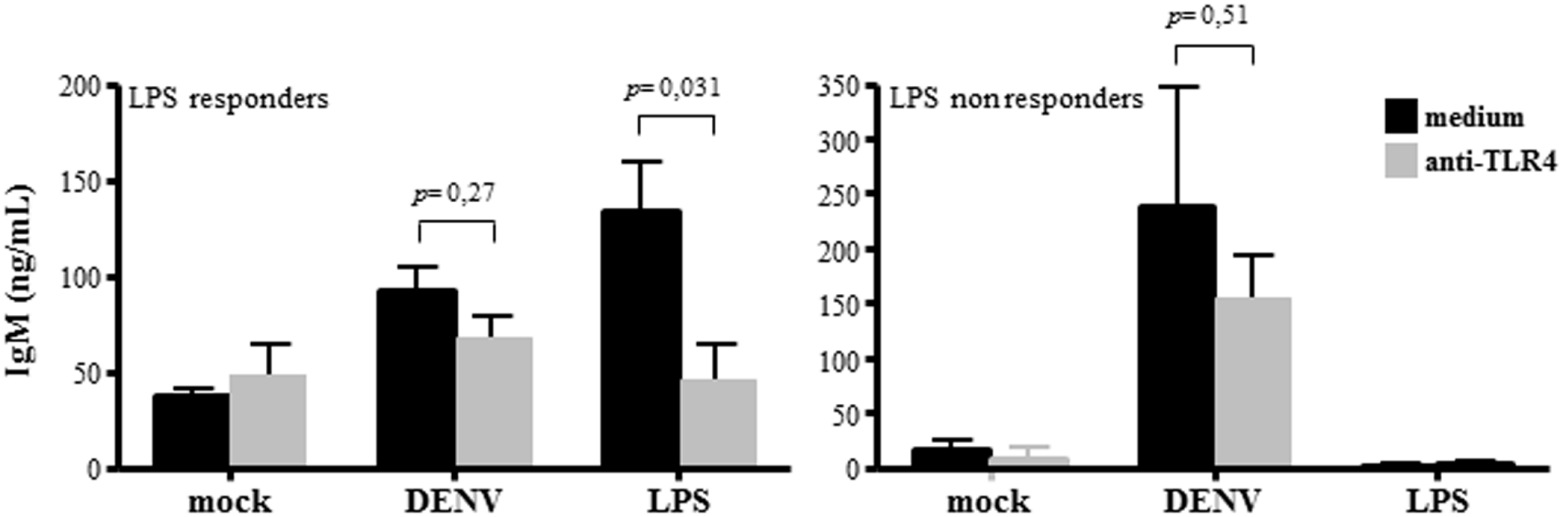
DENV-induced IgM secretion does not depend on TLR4 activation. B lymphocytes were mock-treated or cultured with DENV2 (MOI = 1) in the presence or absence of anti-TLR4 neutralizing antibody. LPS (10μg/ml; Sigma Aldrich) was used as positive control. At 12 days post infection, the supernatants were harvested and IgM levels were measured by ELISA. B cell cultures which showed increased IgM secretion in response to LPS (LPS responders) were graphed separately from the ones that didn’t show (LPS nonresponders). Data are representative of four independent experiments. Statistical analysis were performed and p values are indicated in the figures.

Polyclonal B cell activation is usually associated to TLR engagement and it was recently demonstrated that DENV NS1 activates TLR4 in different cell types [40]. To address whether DENV-induced B cell activation was also dependent on TLR4 activation, B cells were cultured with either DENV or LPS and TLR4 was neutralized by specific antibody. B cells obtained from some donors were not activated by LPS, therefore, we individually analyzed DENV-induced IgM secretion by B cells that were stimulated or not by LPS (LPS responders and nonresponders). As expected, TLR4 neutralization abolish LPS-induced IgM secretion; however, it did not significantly affect IgM secretion stimulated by DENV, indicating that B cell activation by the virus did not depend on TLR4 signaling. Accordingly, increased IgM secretion induced by DENV infection was observed in both LPS responders and nonresponders B cell cultures (Fig 5).

**Fig 6:**
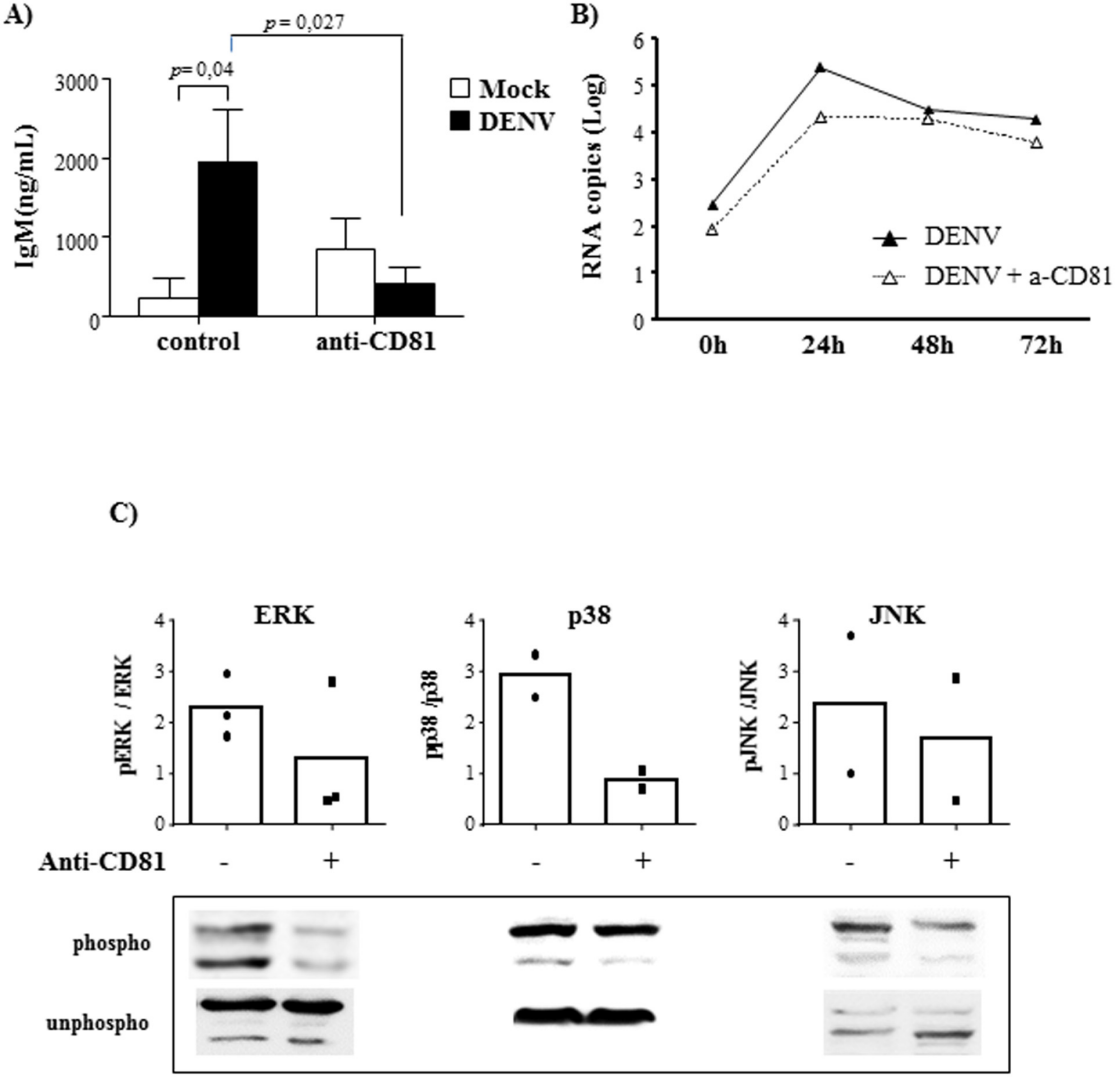
DENV-induced B cell activation depends on CD81 activation. B lymphocytes were mock-treated or cultured with DENV2 (MOI = 1) in the presence or absence of anti-CD81 neutralizing antibody. **A)** At 12 days post infection, the supernatants were harvested and IgM levels were measured by ELISA. Data are representative of four independent experiments. Statistical analysis were performed and p values are indicated in the figures. **B)** The cells were harvested after different time points and DENV RNA levels were evaluated by qRT-PCR. Data are representative of three independent experiments. **C)** After 48h, the cells were harvested and the expression of ERK, p38 and JNK MAPK, phosphorylated (phospho) or not (unphospho) were analyzed in the cell lysates by western blotting, as indicated. The bars indicate the ratio between the analyzed phosphorylated protein and the corresponding unphosphorylated one; dots represent individual data.

Since CD81 was previously characterized as a B cell receptor for HCV Flavivirus, we investigated whether this molecule would also have a role in DENV-mediated B cell activation. Purified B cells were incubated with anti-CD81 neutralizing antibody and then infected with DENV2. CD81 blockage almost abrogated IgM secretion (Fig 6A), indicating that this molecule participates in the activation of B cells by dengue virus. Analysis of DENV RNA in the cells treated with anti-CD81, however, revealed that this receptor was not essential for B cell infection (Fig 6B).

On the other hand, neutralization of CD81 significantly affect MAPK phosphorylation, as observed by western blot analysis of ERK, p38 and JNK phosphorylated/unphosphorylated proteins (Fig 6C), indicating that DENV-induced CD81 activation might promote MAPK phosphorylation, leading to IgM secretion. Importantly, inhibition of IgM secretion mediated by CD81 neutralization or MAPK inhibition were not due to intrinsic cytotoxicity of the antibody nor of the inhibitors, as demonstrated in S4 Fig.

### DENV induces increased expression of costimulatory molecules by B cells, and IgG secretion by PBMCs

**Fig 7:**
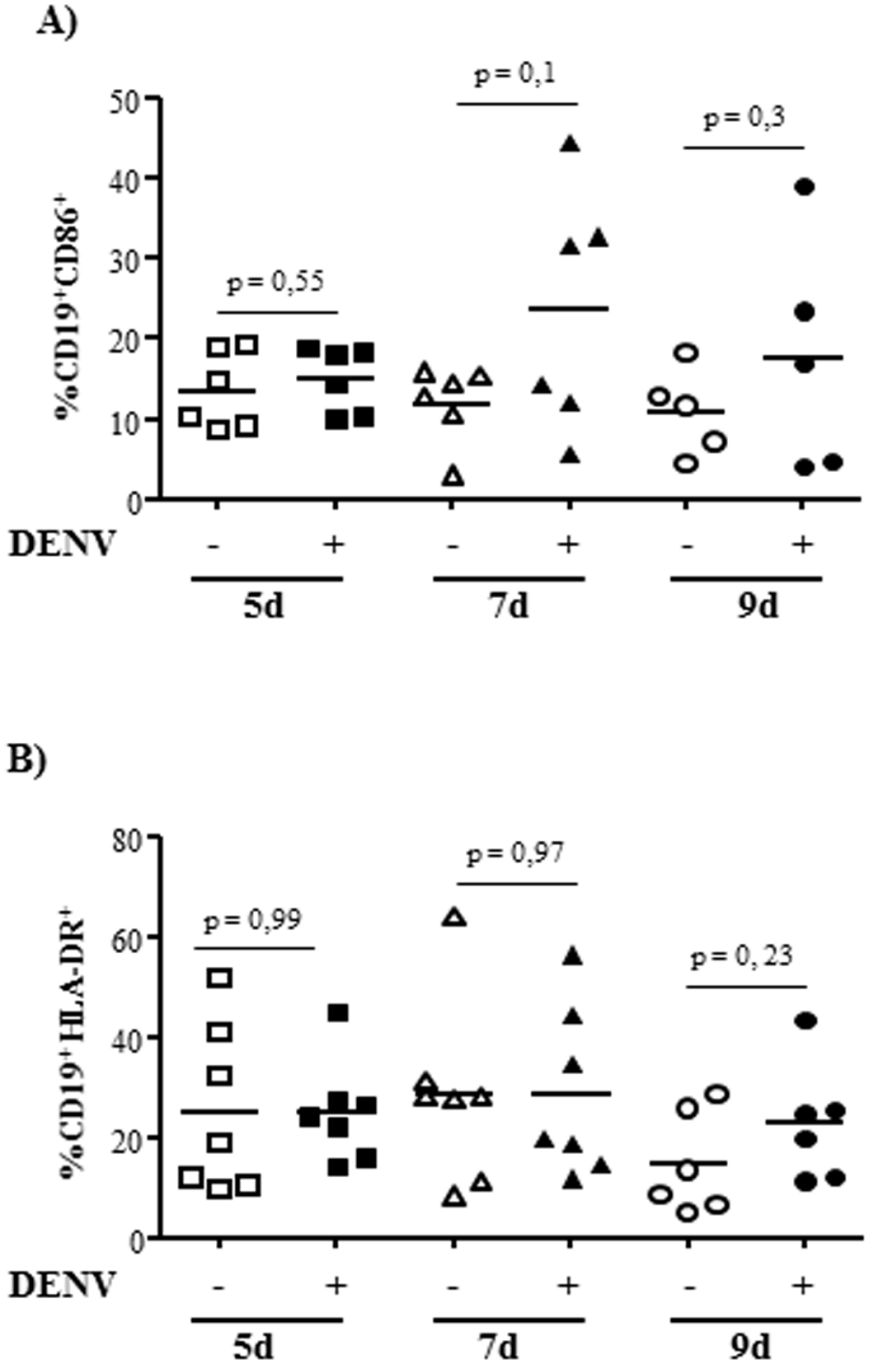
Purified B cells cultured with dengue virus showed increased expression of costimulatory molecules. B lymphocytes were mock-treated or cultured with DENV2 (MOI = 1) for the indicated time points and the expression of CD86 (A) or HLA-DR (B) in CD19+ cells were evaluated by flow cytometry. Each point indicate an individual donor. Statistical analysis were performed and p values are indicated in the figures.

**Fig 8:**
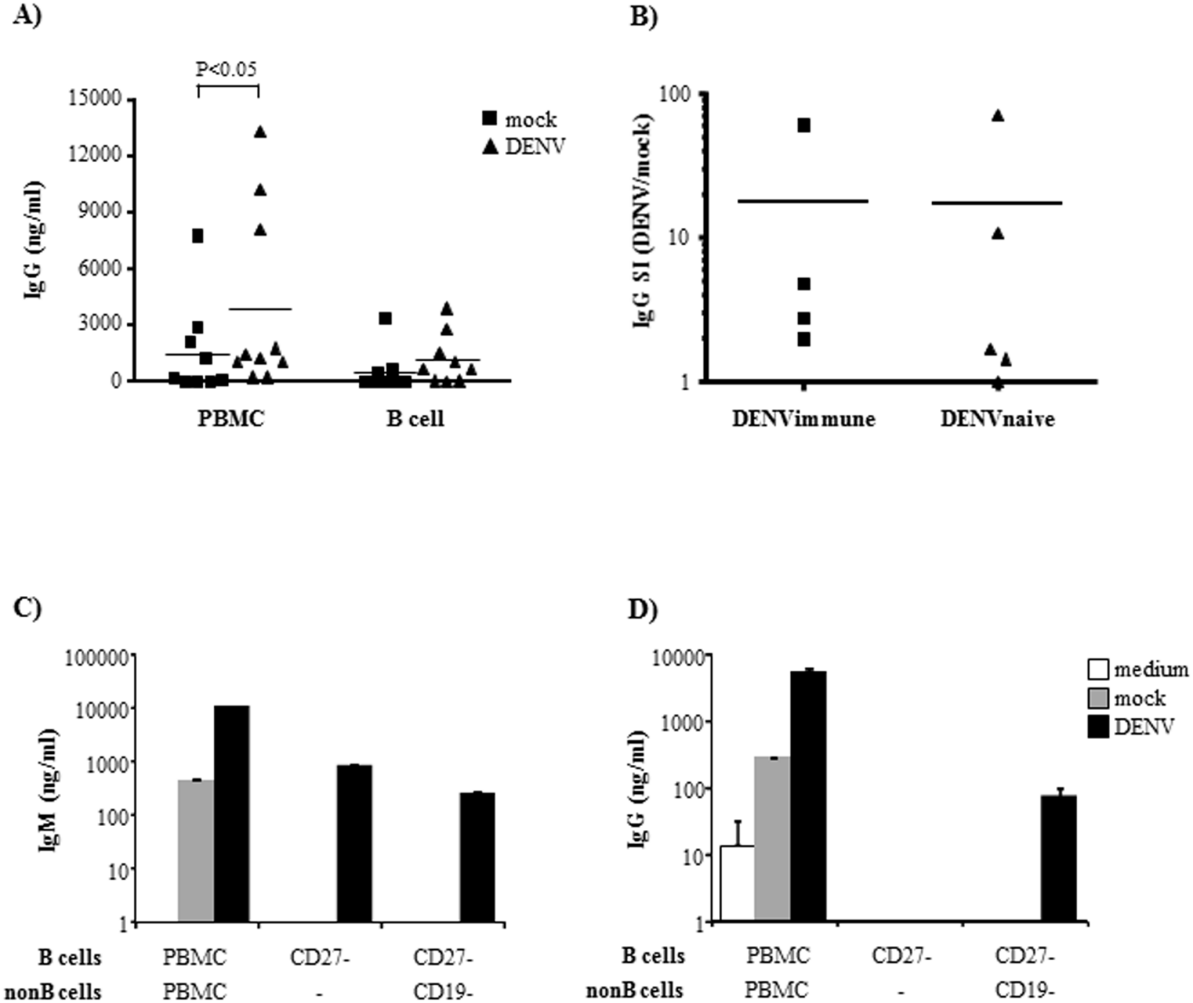
DENV infection of PBMC promotes IgG secretion. **A)** PBMCs or purified B cells were mock-treated or cultured with DENV2. After 12 days p.i., the supernatants were harvested and IgG secretion was evaluated by ELISA. Squares (mock) and triangles (DENV) indicate individual donors. **B)** PBMCs obtained from DENV immune (serum IgG+) and DENV naïve (serum IgG-) donors were mock-treated or cultured with DENV2, and IgM secretion was evaluated after 12 days. Stimulation index was calculated as the ratio between IgM concentration obtained from DENV and mock treated cultures. Dots indicate individual donors. **C-D)** PBMCs, CD3-CD27- cells (naïve Bcells), or CD3-CD27- and CD19+ cells (coculture between naïve B cells and non B cells), from the same donor were mock treated or DENV-infected. After 12 days p.i., the supernatants were harvested and IgM **(C)** or IgG **(D)** secretion were evaluated by ELISA. Bars indicate the average and SD of, at least, two independent experiments.

We also evaluated whether interaction with DENV would affect other cell activation markers, such as expression of costimulatory molecules and Ig class switching. We observed that DENV induced an increased expression of CD86 and HLA-DR in several but not all individuals (Fig 7A and 7B), which may enhance their antigen presentation ability and improve their interaction with other cell types. We then cultured either B cells or PBMCs with the virus and analyzed IgG secretion. We observed that PBMC, but not purified B cells, secreted elevated amounts of total IgG (Fig 8A), indicating that DENV induced Ig class switch in a way dependent on further stimuli by other cell types. This is consistent with previous findings, which demonstrated that DENV-infected monocytes stimulated B cell differentiation into plasmablasts [41].

IgG secretion levels in the B cell cultures obtained from DENV immune or DENV naïve donors were comparable (Fig 8B), indicating that IgG secretion might not be only a consequence of memory B cell activation. To further evaluate this hypothesis, naïve B cells were sorted and cultured with DENV, alone or in the presence of non B mononuclear cells. As previously observed, total PBMCs secreted IgM and IgG when cultured with DENV (Fig 8C and 8D). Infection of isolated naïve B cells promoted IgM, but not IgG secretion; in contrast naïve B cells cultured with other cell types also secreted IgG after DENV infection, indicating that DENV-mediated B cell activation might prime these cells and allow isotype class switch in the presence of other stimulated cell types.

## Discussion

In the present work, we demonstrated that although interaction of primary human B cells with DENV may not result in an efficient productive infection, it directly activates the cells, inducing polyclonal IgM secretion, cytokine production and expression of costimulatory molecules. This study was motivated by the observation that DENV-exposed individuals present abnormally increased levels of circulating activated B cells [33, 34, 35], which might not be explained only by clonal expansion of DENV-specific cells. The characterization of DENV neutralizing antibodies and the description of the phenotype of circulating B cells started to be investigated recently, but the direct role of virus-cell interaction in this activation was not addressed before.

Initially, we analyzed whether DENV could replicate in B cells, since even the permissivity of B lymphocytes to dengue infection was still a controversial issue. We observed that B cells were, in fact, susceptible to dengue; however, although B cells expressed NS1 and released virus RNA, those viral particles were not able to efficiently infect other cells. Earlier studies demonstrated the presence of DENV antigens in the B cell population present in the PBMC from infected patients [42, 43]. In addition, one study demonstrated that B lymphocytes were able to support DENV infection *in vitro*, in a high MOI, suggesting that this cell type could be a target cell for DENV [39]. However, other studies assessing the infection of PBMC and splenocytes showed that only peripheral blood monocytes and splenic macrophages were efficiently infected, while T and B cells were not permissive for DENV even in the presence of immune serum [44, 45]. Although we have demonstrated that the viruses released by B cells are inefficient in infecting permissive cell lines, those defective virus or immature virions may still have a role in dengue pathogenesis. Partially mature virus are known to be target of Ig, forming immunocomplexes, which mediate cell infection via FcγR, and promote the production of infectious progeny. In addition, uncleaved prM protein and fusion loop, which are exposed on immature virus particles are highly immunogenic and are able to trigger an abundant antibody response [13]. Therefore, it would be important to further investigate the relevance of B cell infection for DENV dissemination and immune activation.

Even though B cells might not to be an important element for virus propagation, the fact that the virus binds and is internalized by those cells migth be sufficient to interfere with cell physiology. In fact, we demonstrated that DENV interaction with B cells induced high level of immunoglobulin secretion in way dependent on CD81 and MAPK activation. This effect might not be associated to memory DENV-specific cells activation, since we detected mainly IgM and not IgG in the cultures, unless other PBMCs were present. In addition, increased levels of IgM were detected even in the cultures from DENV-naïve individuals, and were observed after infection of naïve B cells, indicating that it might be a natural non-specific polyclonal B cell activation response induced by the virus. Since we did not use purified virus, we cannot completely discard the hypothesis that other soluble mediators released by infected C6/36 cells might be contributing to B cell activation; however, mock-treated cultures did not activated the cells and Ig secretion was diminished when lower MOI was used in the cultures (data not shown).

Polyclonal activation of B cells was demonstrated in several different viral infections, such as influenza, hepatitis C, and HIV [46, 47, 48], and it is usually related to the secretion of cross-reactive, low affinity antibodies. Interestingly, antibodies produced early during dengue infection were shown to exhibit low specificity and affinity. In fact, high levels of cross-reactive antibodies are observed during both primary and secondary DENV infection, indicating that it might not be exclusively related to virus-specific priming response. The faster and higher cross-reactive response to all four DENV serotypes during secondary infection corroborates the idea that it may represent the activation or re-activation of a polyspecific pool of B cells [34]. Cross-reactivity or polyspecificity of antibodies detected in DENV-infected patients were not only related to the recognition of different virus serotypes, but also to host proteins, including platelets, endothelial cells and plasminogen [49, 50, 51]. In addition, those autoantibodies were associated to complement activation, modulation of platelet aggregation and apoptosis of endothelial cells, and their production may be correlated to the evolution of severe dengue [49, 50, 51, 52, 53]. Our data indicated that direct interaction between DENV and B cells induced a polyclonal B cell activation and might contribute to those events. Polyclonal B cell activation may also represent viral induction of original antigenic sin, which may be a potential immune escape mechanism, as described for T cell in dengue infection [5]. It may also stimulate a competition phenomenon and contributes to B cell exhaustion, resulting in less efficient neutralization and increased risk associated with secondary infections. Corroborating to this hypothesis, it has been reported that although acute DENV infection is associated to increased numbers of plasmablasts, dengue patients present lower absolute plasmablast numbers at convalescence, in comparison to control patients [54].

Activation of B cells and increased immunoglobulin secretion mediated by CD81 activation was also reported during HCV infection. In this model, CD81-mediated Ig hypermutation was associated to a decreased antibody affinity to E2 protein and lowered neutralizing activity [55]. Therefore, DENV-CD81 interaction might result in the induction of poly-specific, cross-neutralizing and subneutralizing antibodies observed in dengue patient’s sera, which might contribute to viral antigenic sin, exhaustion and inefficient response in a secondary infection.

The role of CD81 for B cell stimulation is intriguing, since this molecule is not usually associated to intracellular signaling itself. In most of the systems, CD81-mediated B cell activation is dependent on its tetraspanin function, which is associated to the membrane expression and crosslink of CD19 or BCR molecules [56, 57]. However, CD81 engagement by HCV-E protein was associated to B cell activation and Ig somatic hypermutation, in a way independent on BCR or CD19 coligation [31, 32]. In HCV infection, CD81 participates in virus entry [58], which did not seem to be the case in our system. However, the role of virus entry for B cell activation had not been directly investigated during HCV-B cell interaction. Interestingly, CD81 engagement by specific antibodies in healthy naïve B cells had been associated to B cell proliferation and expression of activation markers, such as CD69 and C86 [32], supporting the idea that this molecule can function as a B cell activation receptor. Still, whether CD81 is a DENV activation receptor itself, or if it is part of a complex, or even, whether its tetraspanin function is needed to induce crosslink of other B cell receptors requires further investigated.

Finally, B cells presented enhanced expression of HLA-DR and CD86, which might help in the interaction and activation of other cell types. In turn, PBMCs stimulated by DENV can secrete increased levels of IgG, demonstrating that other cell types might potentiate B cell activation, resulting in increased IgG levels *in vivo*. This is supported by previous findings, which demonstrated that CD14^+^CD16^+^ monocyte infected by DENV could induce plasmablast differentiation and IgG secretion in vitro [41].

In conclusion, our data support the idea that dengue virus directly interact with B lymphocytes, triggering signaling activation pathways that mediates Ig and cytokine secretion. The secretion of high amount of IgM *in vitro* even by cells isolated from DENV naïve donors indicates that virus-cell interaction might not depend on BCR specificity, what might contribute to the substantial numbers of activated B cells and cross-reactive plasma Ig observed in patients. Further analysis of the receptors and signaling pathways associated to DENV-mediated polyclonal Ig secretion may therefore help in unveiling the role of B lymphocytes in DENV pathogenesis.

## Supporting Information

S1 Fig Sorting strategy for separation of naïve B cells and non B cells. PBMCs were cultured for 2h for cell adhesion; then, non adherent cells were incubated with PE-anti-CD19 antibody or with PE-anti-CD27 and FITC-anti-CD3. **A)** Sorting strategy for separation of CD19 negative cells (non B). **B)** Sorting strategy for separation of CD3negative, CD27negative cells (naïve B cells)
(TIF)

S2 Fig Activation of human B cells by DENV1. Purified B lymphocytes were mock-treated or cultured with DENV1 at a MOI of 1. After 12 days p.i., the supernatants were harvested and IgM levels were measured by ELISA. The lines indicate the IgM response to mock-treatment or DENV infection obtained from the same donor.
(TIF)

S3 Fig B cell infection by DENV does not affect the pattern of total tyrosine nor AKT phosphorylation. B lymphocytes were mock-treated or cultured with DENV2 (MOI = 1). A) The cells were harvested after 48h p.i., and the expression of phosphotyrosine were analyzed in the cell lysates by western blotting. The cells were also stained with anti-βactin antibody as a loading control. B) The cells were harvested after 2h or 48h p.i., and the expression of phosphorylated (pAKT) or unphosphorylated AKT (AKT) were analyzed in the cell lysates by western blotting, using the indicated antibodies. Bars indicate the ratio between the analyzed phosphorylated protein and the corresponding unphosphorylated one. Data are representative of two independent experiments.
(TIF)

S4 Fig Evaluation of the cytotoxicity of anti-CD81 and MAPK inhibitors in B cell cultures. A) B lymphocytes were cultured with DENV2 (MOI = 1) in the presence or absence of ERK (PD98059), p38 (SB203580) and JNK (SP600125) inhibitors, or anti-CD81 antibody. After 72h, the cells were incubated with PI and analyzed by flow cytometry. B) B lymphocytes were cultured with anti-CD81 antibody at different concentrations and, after 72h, cell viability was evaluated by XTT assay. C) B cells were mock-treated or cultured with DENV in the presence or absence of anti-CD81. After 72h, the supernatants were harvested and the amount of released lactated dehydrogenase (LDH) was evaluated, as described.
(TIF)
