# Supplementary figures and images for: Dengue Virus Directly Stimulates Polyclonal B Cell Activation
Source: PLoS One. 2015 Dec 10;10(12):e0143391. doi: 10.1371/journal.pone.0143391 (PMC4675537; doi:10.1371/journal.pone.0143391)

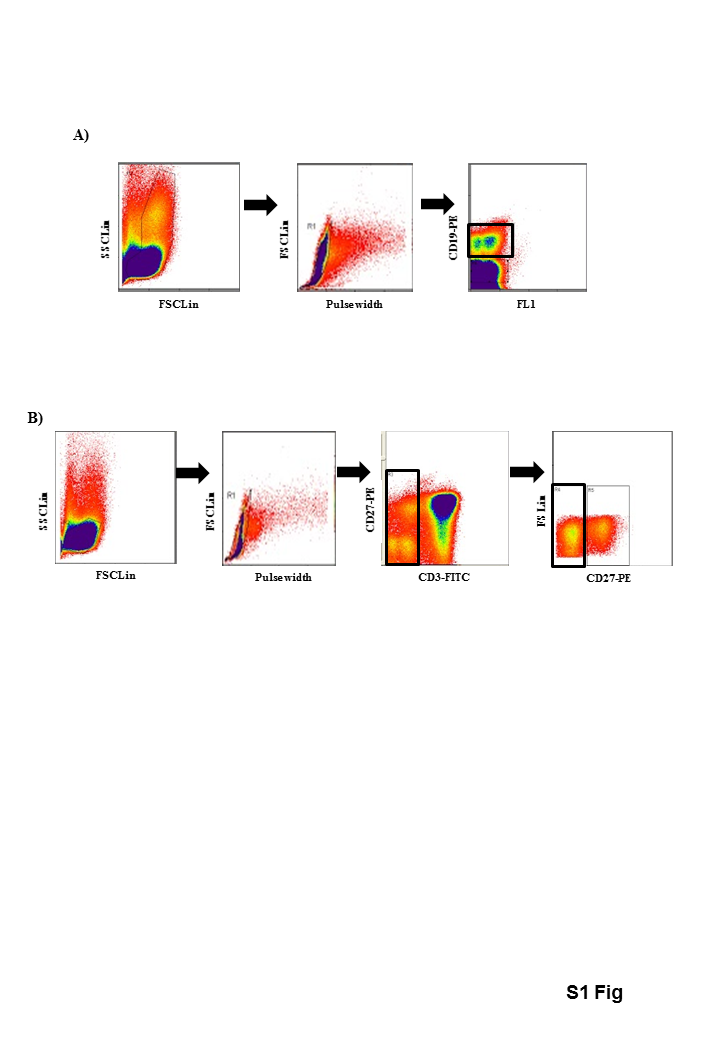

Supplement: S1 Fig — PBMCs were cultured for 2h for cell adhesion; then, non adherent cells were incubated with PE-anti-CD19 antibody or with PE-anti-CD27 and FITC-anti-CD3. A) Sorting strategy for separation of CD19 negative cells (non B). B) Sorting strategy for separation of CD3negative, CD27negative cells (naïve B cells) (TIF) [file pone.0143391.s001.tif]

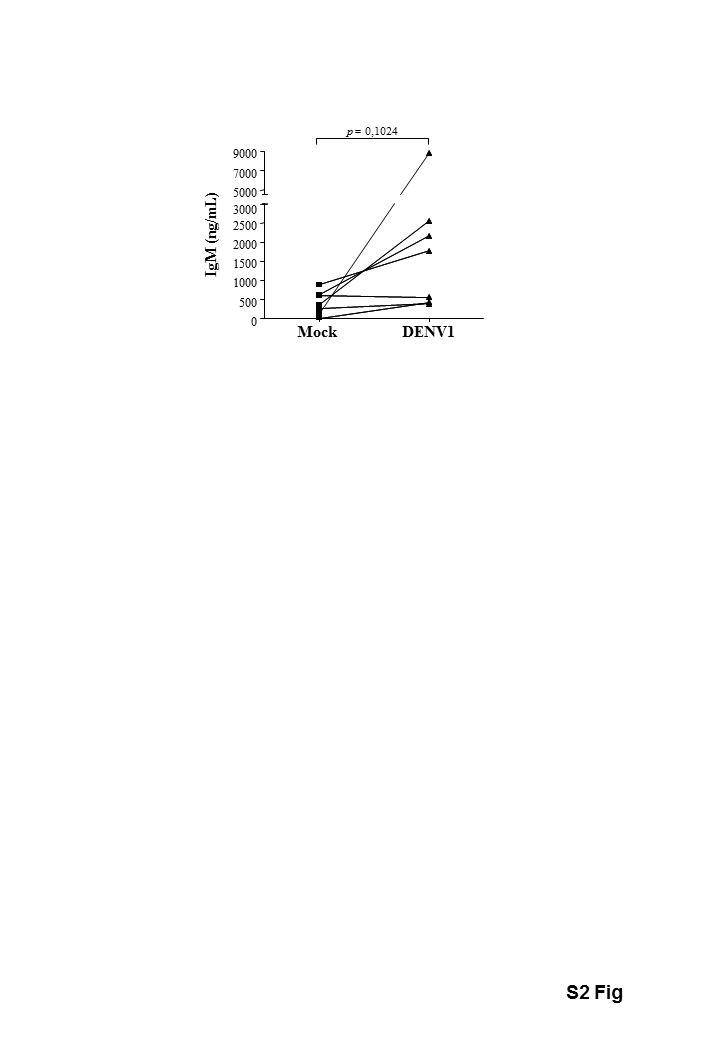

Supplement: S2 Fig — Purified B lymphocytes were mock-treated or cultured with DENV1 at a MOI of 1. After 12 days p.i., the supernatants were harvested and IgM levels were measured by ELISA. The lines indicate the IgM response to mock-treatment or DENV infection obtained from the same donor. (TIF) [file pone.0143391.s002.tif]

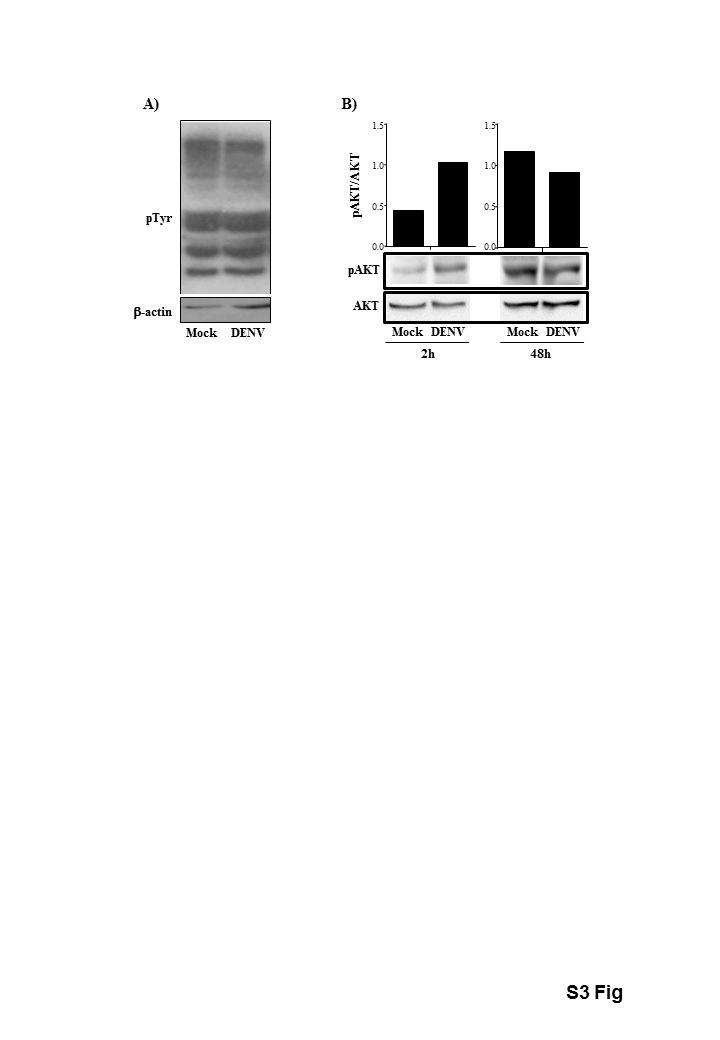

Supplement: S3 Fig — B lymphocytes were mock-treated or cultured with DENV2 (MOI = 1). A) The cells were harvested after 48h p.i., and the expression of phosphotyrosine were analyzed in the cell lysates by western blotting. The cells were also stained with anti-βactin antibody as a loading control. B) The cells were harvested after 2h or 48h p.i., and the expression of phosphorylated (pAKT) or unphosphorylated AKT (AKT) were analyzed in the cell lysates by western blotting, using the indicated antibodies. Bars indicate the ratio between the analyzed phosphorylated protein and the corresponding unphosphorylated one. Data are representative of two independent experiments. (TIF) [file pone.0143391.s003.tif]

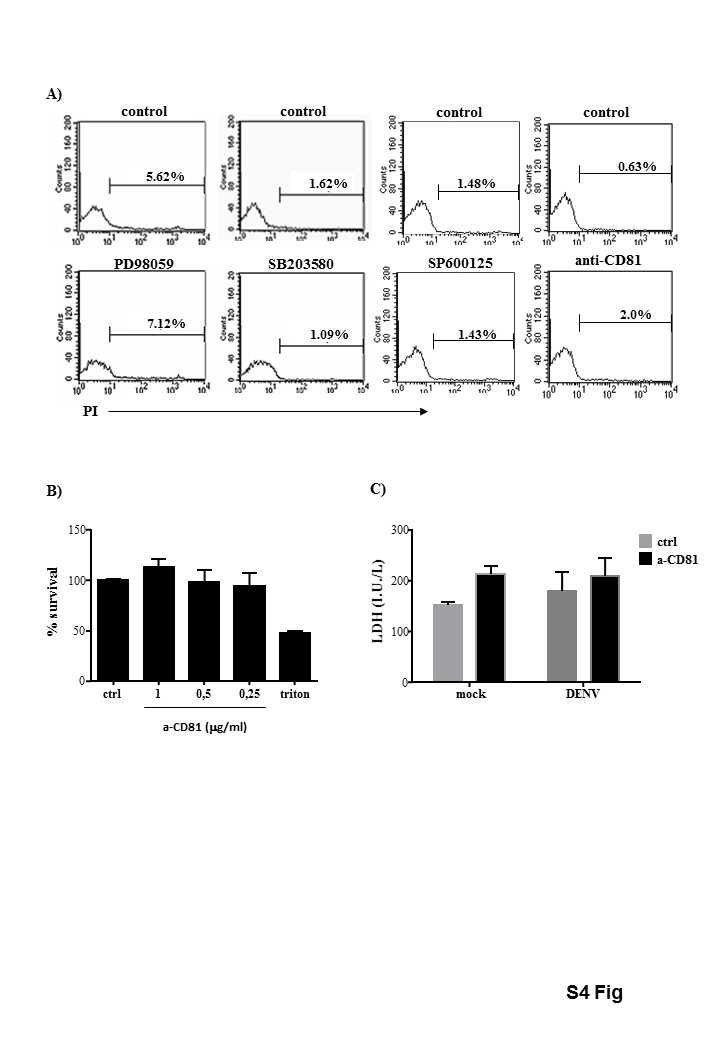

Supplement: S4 Fig — A) B lymphocytes were cultured with DENV2 (MOI = 1) in the presence or absence of ERK (PD98059), p38 (SB203580) and JNK (SP600125) inhibitors, or anti-CD81 antibody. After 72h, the cells were incubated with PI and analyzed by flow cytometry. B) B lymphocytes were cultured with anti-CD81 antibody at different concentrations and, after 72h, cell viability was evaluated by XTT assay. C) B cells were mock-treated or cultured with DENV in the presence or absence of anti-CD81. After 72h, the supernatants were harvested and the amount of released lactated dehydrogenase (LDH) was evaluated, as described. (TIF) [file pone.0143391.s004.tif]
